# Supplementary material for: Predicting the Potential Distribution of Hypericum perforatum under Climate Change Scenarios Using a Maximum Entropy Model
Source: Biology (Basel). 2024 Jun 19;13(6):452. doi: 10.3390/biology13060452 (PMC11201051; doi:10.3390/biology13060452)
Supplement: Supplementary file 1 [file biology-13-00452-s001.zip › Figure S1.pdf]

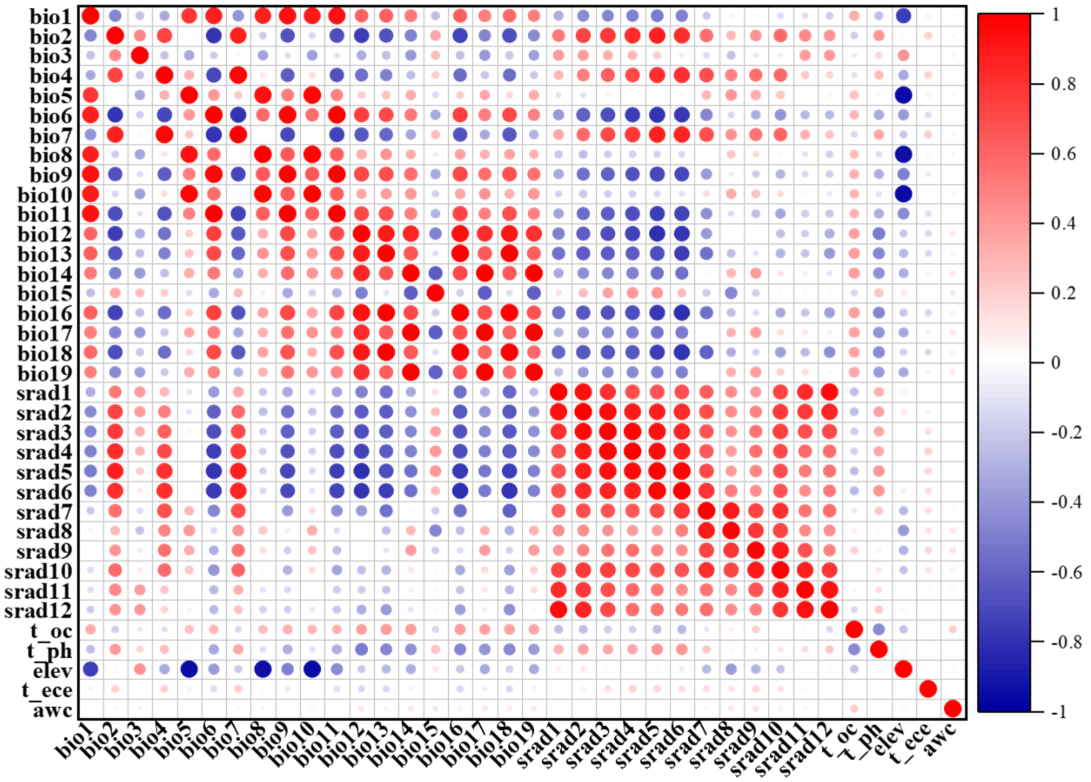

Figure S1. Correlation analysis of various environmental factors. Red representspositive correlations, and blue represents negative correlations. The deep red and blue representhigher correlation coefficients between two variables.
