# Supplementary material for: Predicting the Potential Distribution of Hypericum perforatum under Climate Change Scenarios Using a Maximum Entropy Model
Source: Biology (Basel). 2024 Jun 19;13(6):452. doi: 10.3390/biology13060452 (PMC11201051; doi:10.3390/biology13060452)
Supplement: Supplementary file 1 [file biology-13-00452-s001.zip › Table S2.pdf]

Table S2. Description of environmental variables used in MaxEnt.

Num.	Variable	Connotation	Unit
1	Bio1	Annual mean temperature	℃
2	Bio2	Mean diurnal range	℃
3	Bio3	Isothermality	-
4	Bio4	Temperature seasonality	-
5	Bio5	Max temperature of warmest month	℃
6	Bio6	Min temperature of coldest month	℃
7	Bio7	Temperature annual range	℃
8	Bio8	Mean temperature of wettest quarter	℃
9	Bio9	Mean temperature of driest quarter	℃
10	Bio10	Mean temperature of warmest quarter	℃
11	Bio11	Mean temperature of coldest quarter	℃
12	Bio12	Annual precipitation	mm
13	Bio13	Precipitation of wettest month	mm
14	Bio14	Precipitation of driest month	mm
15	Bio15	Precipitation seasonality	-
16	Bio16	Precipitation of wettest quarter	mm
17	Bio17	Precipitation of driest quarter	mm
18	Bio18	Precipitation of warmest quarter	mm
19	Bio19	Precipitation of coldest quarter	mm
20	Srad1	January solar radiation	kJm-2day-1
21	Srad2	February solar radiation	kJm-2day-1
22	Srad3	March solar radiation	kJm-2day-1
23	Srad4	April solar radiation	kJm-2day-1
24	Srad5	May solar radiation	kJm-2day-1
25	Srad6	June solar radiation	kJm-2day-1
26	Srad7	July solar radiation	kJm-2day-1
27	Srad8	August solar radiation	kJm-2day-1
28	Srad9	September solar radiation	kJm-2day-1
29	Srad10	October solar radiation	kJm-2day-1
30	Srad11	Solar radiation in November	kJm-2day-1
31	Srad12	Solar radiation in December	kJm-2day-1
32	Elev	Elevation	m
33	Awc	Soil available water content	1
34	t_pH	Soil pH	1
35	t_oc	Soil organic carbon content	%
36	t_ece	Soil electrical conductivity	%
